# Larches of Kuzhanovo Have a Unique Mutation in the *atpF–atpH* Intergenic Spacer

**DOI:** 10.3390/ijms24043939

**Published:** 2023-02-15

**Authors:** Alexander Artyukhin, Yuliya Sharifyanova, Mikhail M. Krivosheev, Elena V. Mikhaylova

**Affiliations:** 1 Institute of Biochemistry and Genetics UFRC RAS, Prospekt Oktyabrya 71, Ufa 450054, Russia; 2 Faculty of Biology, Bashkir State University, Zaki Validi 32, Ufa 450076, Russia

**Keywords:** *Larix sibirica*, *Larix sukaczewii*, polymorphism, *atpF–atpH*, rpoC1, ploidy

## Abstract

The larches of Kuzhanovo (*Larix sibirica* Ledeb.) are protected trees with a round crown growing in the Southern Urals. In 2020 vandals sawed the sapwood of these trees, which exposed the problem of insufficient conservation measures. Their origin and genetic characteristics have been of particular interest to breeders and scientists. The larches of Kuzhanovo were screened for polymorphisms using SSR and ISSR analyses and the sequencing of genetic markers and genes *GIGANTEA* and *mTERF*, associated with wider crown shape. A unique mutation was discovered in the *atpF–atpH* intergenic spacer of all protected trees, but it was absent in some of their descendants and larches with similar crown shape. Mutations were discovered in the *rpoC1* and *mTERF* genes of all samples. Flow cytometry did not reveal any changes in genome size. Our results suggest that the unique phenotype arose from point mutations in *L. sibirica*, but they are yet to be found in the nuclear genome. The concurrent mutations in the *rpoC1* and *mTERF* genes may indicate that the round crown shape originates from the Southern Urals. The *atpF–atpH* and *rpoC1* genetic markers are not common in studies of *Larix* sp., but their wider use could help to establish the origin of these endangered plants. The discovery of the unique *atpF–atpH* mutation also allows for stronger conservation and crime detection efforts.

## 1. Introduction

Larches are rarely considered ornamental trees, and are mainly valued for their high-quality wood. Nevertheless, remarkable cold tolerance favorably distinguishes larches from deciduous trees, and among conifers they are notable for their autumn color [1]. Trees with unusual phenotypes such as weeping crown or witch brooms have always drawn the attention of people and have been used as landscape plants. However, these traits usually cannot be inherited, and require micropropagation.

The larches of Kuzhanovo are eleven protected trees growing in a limited area in the Abzelilovsky District of The Republic of Bashkortostan, Southern Urals. They have an unusual ornamental round crown, more characteristic of *Preudloarix amabilis* and *Araucaria bidwillii* than *Larix* sp. Most of these trees appear to be more than one hundred years old [2]. There are several legends about their origin, but they have never been confirmed by genetic studies. According to phenotypic characteristics, these plants are considered to be *Larix sukaczewii* Dylis (*L. sibirica*). Unlike plants with weeping or witch broom phenotype caused by somatic mutations and infections, the larches of Kuzhanovo pass on the shape of their crown to a small proportion of offspring. It is important to note that the seed yield of the larches of Kuzhanovo is very low, and only a small fraction of descendants inherit the shape of the crown. Discovery of the genetic marker associated with the desired trait provides an opportunity to propagate new trees from seeds and use them for landscaping universally.

Public attention was drawn to the larches of Kuzhanovo in 2020, when vandals sawed the sapwood of these trees, which exposed the problem of insufficient conservation measures. One of the trees did not survive, but others were recovered by closing the cuts using donor vascular tissues and plastic wrap. Further studies have shown that the trunks of two trees were affected by rot [2]. Therefore, genetic studies of these unique trees are extremely important not only for conservation of the larches of Kuzhanovo, but also for gardening and landscaping.

The round crown shape could be a result of mutations of various levels or hybridization. Spontaneous hybridization and mixoploidy have been documented in *Larix* species [3,4,5]. They can affect larch growth parameters and induce a large genetic variability [1,6]. Full chloroplast genomes of several *Larix* species have been sequenced, which makes it possible to determine the species of the larches of Kuzhanovo and the presence of genetic polymorphisms. Sequencing of the chloroplast markers has been widely used in *Larix* sp. [7,8]. Nuclear markers such as internal transcribed spacers (ITS) appeared to be quite polymorphic between populations of *L. sukaczewii* and *L. sibirica* in [9]. Several other methods, such as RAPD, AFLP, ISSR and SSR analyses, were also used to study genetic polymorphism in larches [5,10,11].

Mutations directly associated with crown shape in *Larix* sp. are still unknown because the nuclear genomes of larches are large and hard to assemble. There are very limited data on several candidate genes associated with abnormal crown morphology in *Picea abies* L., such as circadian clock gene *GIGANTEA*, *AP2L3* and mitochondrial transcription termination factor-related *mTERF* [12]. Mutations in *AP2L3* were abundant in trees with intermediate crown shape; however, mutations in *mTERF* and *GIGANTEA* were observed in higher proportions in the broad-crowned trees. Therefore, these two genes might be of particular interest. The genomes of *Larix sibirica* and *Larix kaempferi* have been sequenced, but knowledge of individual genes in larches remains very limited [13,14,15]. Homologs of these candidate genes have not yet been identified in *L. sibirica*.

In the present study, the larches of Kuzhanovo were screened for polymorphisms using SSR and ISSR analyses and sequencing of genetic markers and candidate genes. Ploidy level was determined via flow cytometry.

## 2. Results

### 2.1. Flow Cytometry

**Figure 1:**
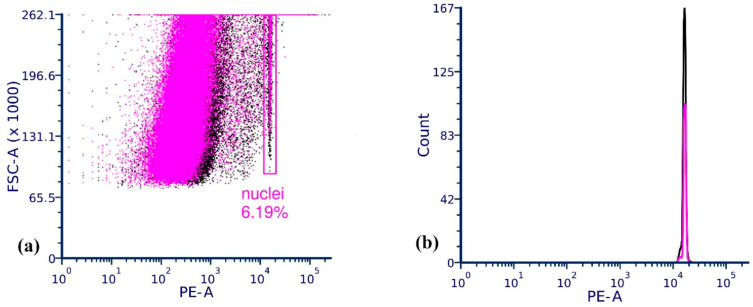
Flow cytometric histograms showing the overlay of nuclei profiles in the larches of Kuzhanovo (pink) and normal larches (black). Nuclei were gated on an *FSC-A* plot (**a**) and their fluorescence intensities were compared (**b**).

According to the results of flow cytometry, there were no signs of changes in the DNA content, abnormal chromosome numbers or genome duplication in the larches of Kuzhanovo, which suggests that these plants did not undergo structural chromosome mutations or hybridization (Figure 1). A single peak was observed on all histograms built for all samples.

### 2.2. SSR and ISSR Analyses

While ISSR markers (CA)_6_GT and (AGC)_6_G were reported to be polymorphic in populations of *L. sibirica* [16], the larches of Kuzhanovo demonstrated the same pattern as control plants. Three polymorphic bands were amplified in the larches of Kuzhanovo with primer (GTG)_5_, originally used for differentiation of *L. kaempferi* [17]. In larches with a round crown growing outside of the protected area, these bands were mostly absent. This primer also revealed several polymorphisms inside groups (Table 1).

**Figure 2:**
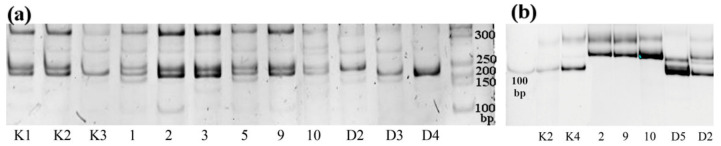
Polyacrylamide gel images showing PCR products amplified by SSR markers Lar_eSSR69 (**a**) and bcLK056 (**b**) of the larches of Kuzhanovo (trees No. 1–10), control plants (K1–4) and larches with a round crown growing at a distance (D1–5).

SSR markers Lar_eSSR11, Lar_eSSR54, Lar_eSSR78, Lar_eSSR96, Lar_eSSR111, Lar_eSSR115, Lar_eSSR228 bcLK232, bcLK260 and bcLK235 did not allow for the detection of any differences between samples. Markers Lar_eSSR69, bcLK224 and bcLK056 were polymorphic, but there was not a single band that could be associated with round crown shape (Figure 2). Nevertheless, marker bcLK056 can be used for identification of the protected trees.

### 2.3. Analyses of Genetic Markers

**Figure 3:**
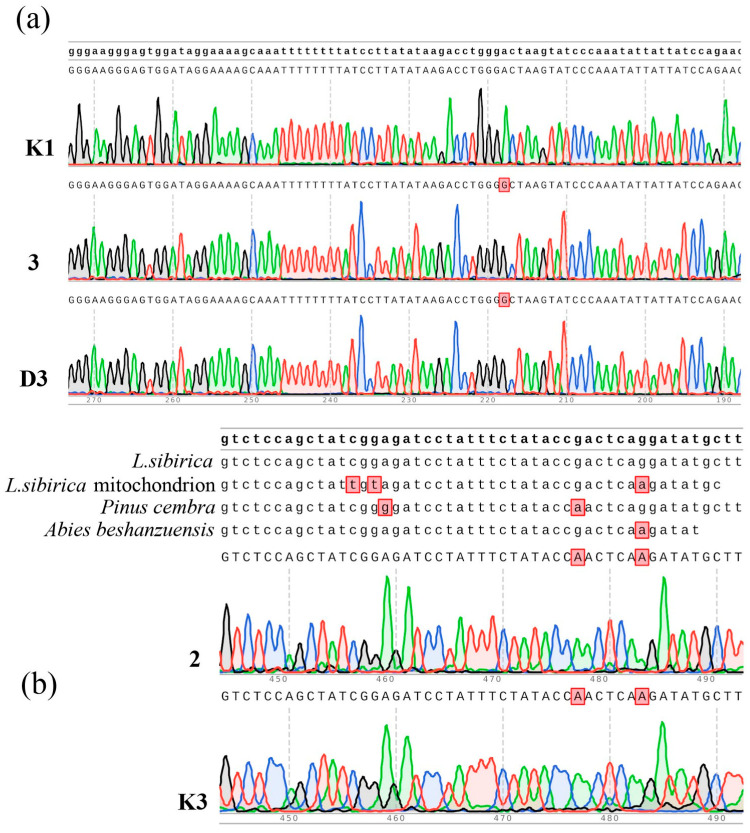
Sequences of the chloroplast *atpF–atpH* region (**a**) and rpoC1 region (**b**) of the larches of Kuzhanovo (trees No. 3 and 2), control plants (K1, K3) and a larch with a round crown growing at a distance (D3).

Sequences of the ITS nuclear region, as well as trnT-trnF, trnK, psbK-psbI, rbcL and rpoC1 chloroplast regions, were not polymorphic in all studied samples. By the nucleotide sequence of the rbcL marker, the samples were closer to *L. kaempferi*, but sequencing of the ITS and trn markers allowed clear identification of all the studied samples as *L. sibirica* (*L. sukaczewii*). However, rpoC1 and psbK-psbI markers do not appear to be variable among species of larches; plants from The Republic of Bashkortostan had two SNPs in the *rpoC1* gene (Figure 3b). One of these mutations was present only in *Pinus* sp., and the second was characteristic of *Tsuga* sp., *Abies* sp., *Cedrus* sp. and *P. amabilis.* It was discovered only in three shotgun sequences of larch genomes, including *L. kaempferi* from China (WOXR02006069.1), *L. cajanderi* (VFAJ01001401.1) from Siberia and *L. sibirica* (NWUY0104493101; MT797191.1) from Krasnoyarsk region [13]. It is interesting that in *L. cajanderi* and *L. sibirica* these mutations were a part of plastid-derived DNA sequences in the mitochondrial genome that did not contain a full *rpoC* gene [18]. In our samples, the *rpoC1* gene with mutations was fully present. 

A unique mutation was also detected In the *atpF–atpH* intergenic spacer of the larches of Kuzhanovo and their progeny growing inside the protected area (Figure 3a). A descendant with a round crown, growing on the private territory, as well as four of the five larches with a round crown growing at a distance, did not carry the mutation.

### 2.4. Analyses of Candidate Genes

The *mTERF* gene appeared to be readily amenable to analysis because in both *P. abies* and *L. sibirica* it consists only of one exon, which is 1413 bp long. According to the NCBI database, in *P. abies* and *L. sibirica* *mTERF* differs by 72 amino acids. Another four mutations were discovered in the studied samples. G/A and A/T substitutions in positions 643 and 734 resulted in amino acid change (V to I, Y to F), however, C/T and A/C mutations did not. Nevertheless, these mutations were not unique for the larches of Kuzhanovo, and were also discovered in several control plants with a normal crown. 

In *P. abies, GIGANTEA* is a gene with two large introns and fourteen exons with a total size of around 65,000 bp It contains duplicated sequences (such as exons 3–5 and 9–11). However, in shotgun sequences of *L. sibirica*, large introns and half of the exons were not present. There were also 76 amino acid substitutions and an 18 bp deletion, as compared to *P. abies*. The amplified product covering exons 13 and 14 was 1672 bp long, but there were no mutations in this region of the studied samples.

## 3. Discussion

While the ISSR and SSR markers used in our study were recommended for distinguishing between populations of larches, they appeared not to be reliable for the identification of trees with a round crown shape. It must be taken into account that contamination of the plant material with infectious agents can interfere with ISSR and SSR methods [19]. 

Chloroplast genomes of larches appear to be rather variable. Among chloroplast markers, *petN-rpoB*, *rps19-rpl2*, *rps14-psbZ*, *psbB-psbN*, *psbD-chlL*, *ccsA-rpl32*, *rps7-ycf2*, *psbI-atpE*, *rbcL-accD*, *petA-psbJ*, *petL-petG* and *rps12-clpP* were polymorphic among five species [7]. In *Larix kaempferi* 25 chloroplast markers, including *psbE*, *psbK*, *rpoC1*, *rpoC2*, *trnS-trnT* and *atpF-ψndhK*, were polymorphic [20]. *trnK* and *trnT-trnF* regions were successfully used for the reconstruction of phylogenetic relationships of *L. sukaczewii* and 12 species of larches [8,21]. Additionally, *5.8S rDNA* including two ITS spacers was studied in *L. sukaczewii* and *L. sibirica* along with structural genes encoding *Cinnamyl alcohol dehydrogenase (CAD)* and *phytochrome-O (PHYO)* [9]. In studied samples, *trnT-trnF*, *trnK* and ITS regions appeared to be monomorphic. Regions *psbK-psbI*, *rbcL*, *atpF–atpH* and *rpoC1* were studied in *L. sibirica* for the first time. Among them, SNPs were discovered only in *atpF–atpH* and *rpoC1*. 

The presence of the same unique mutations in the *rpoC* and *mTERF* genes in larches of The Republic of Bashkortostan suggests that mutations responsible for the crown shape could appear in the Southern Urals, despite the theories of the foreign origin of the larches of Kuzhanovo. The absence of mutation in the *atpF–atpH* region in plants with the round crown outside of the protected area is of particular interest. Pollen, transmitting the chloroplast genome in conifers, is more likely to travel far. However, these trees can only be maternal descendants derived from foreign pollen.

*mTERFs* are key regulators of organellar gene expression. A single mutation in position 566 was associated with a broad-shaped crown in *P. abies* [12]; however, in our study, four new mutations responsible for two amino acid substitutions were also detected in control plants. Therefore, in larches, mutations in this gene are not associated with phenotypic effects.

For the first time we report the possibility of the application of *rpoC* and *atpF–atpH* markers in distinguishing between populations of *L. sibirica*. The *mTERF* gene also appeared to be polymorphic and suitable for phylogenetic studies; however, *GIGANTEA* was conserved. Unique mutation in the *atpF–atpH* region in the larches of Kuzhanovo cannot be directly associated with the crown shape, but this knowledge can help to identify their progeny and facilitate further studies of their origin. 

The lack of prior research studies on the larch genomes and genes involved in crown formation in trees is the most serious limitation of the study. Wider representation of larch genome and gene sequences in databases will help to determine the origin of the larches of Kuzhanovo by detecting the same mutations on other territories. The mutation responsible for the ornamental crown shape is yet to be found in the nuclear genome. Full genome sequencing could help to find the target gene, which we were unable to do with the methods described above. The discovery of such a gene would stimulate the creation of ornamental trees via genetic engineering and genome editing. Nevertheless, ISSR primer (GTG)_5_, SSR primer bcLK056 and sequencing of the *atpF–atpH* region can be used in complex studies to identify the protected larches of Kuzhanovo in a DNA fingerprinting assay. Analysis of the DNA extracted from the plant residues on the saw or axe of the suspects will allow investigators to assess the likelihood of their involvement in the crime. Our findings may help to punish the criminals who sawed the sapwood of the larches of Kuzhanovo, to prevent future attacks and to strengthen the conservation of these unique trees.

## 4. Materials and methods

### 4.1. Plant Material

**Figure 4:**
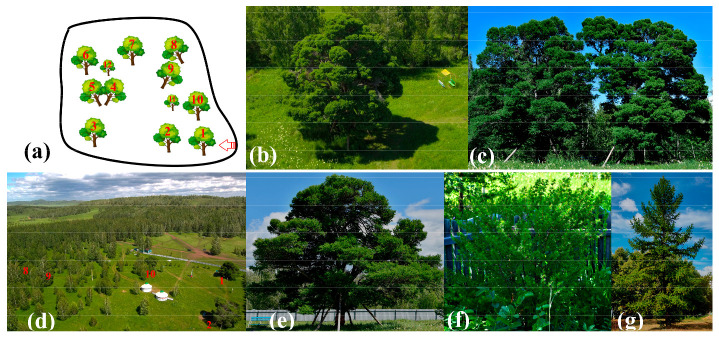
Larches of Kuzhanovo: (**a**) map of the protected territory based on satellite images; (**b**) tree No. 3; (**c**) trees 4 and 5; (**d**) trees 1, 2, 8, 9 and 10; (**e**) tree No. 1; (**f**) descendant No. 11; (**g**) normal larch.

The larches of Kuzhanovo are located on a territory of 16 hectares at the coordinates 53.447598, 58.526692 in the Abzelilovsky District of The Republic of Bashkortostan (Figure 4a,d). Samples were taken from all ten surviving plants and their three descendants. Two descendants are located inside the protected area, and the third grows in Kuzhanovo village, on private territory (Figure 4b–f). Five larches with a similar round crown were discovered in the Abzelilovsky District, outside of the protected area, and used for genetic analysis. Their location is not disclosed for reasons of their safety. In general, all 18 known trees with a round crown were analyzed. Larches with a normal crown shape from Abzelilovsky District, Tatyshlinsky District and the city of Ufa were used as controls (Figure 4g). Sequences were compared to 48 chloroplast genomes of larches *L. sibirica* (NC_036811.1), *L. gmelinii* (MK468648, MK468646, MK468639, MK468638, MK468637, MK468636, MK468635, MK468634, MK468633, MK468632, MK468631, NC_044421, MF990370, LC228572, LC228571, LC228570), *L. cajanderi* (MK468645, MK468644, MK468643, MK468641, NC_044422), *L. potaninii* (KY885247, KX880508, NC_061649, MN822885), *L. kaempferi* (MF990369, LC574976, LC574975, LC574974, LC574973, LC574972, LC574971, LC574970, LC574969), *L. occidentalis* (NC_039583, FJ899578), *L. griffithii* (NC_061650, NC_061646, MN822886, MN822882), *L. kongboensis* (NC_061648, MN822884), *Larix himalaica* (NC_061647, MN822883), *L. decidua* (AB501189, AB547951) and 22 whole-genome shotgun sequences of *L. sibirica* (NWUY0000000000), *L. kaempferi* (WOXR02000000, BSBM00000000), *L. gmelinii* (VFBA01000000, VFAZ01000000, VFAY01000000. VFAX01000000, VFAW01000000, VFAV01000000, VFAU01000000, VFAT01000000, VFAS01000000, VFAR01000000, VFAQ01000000, VFAP01000000, VFAO01000000, VFAN01000000, VFAM01000000, VFAL01000000, VFAK01000000, VFAJ01000000, VFAI01000000).

### 4.2. Ploidy Analysis

Nuclei were extracted using a razor blade in an ice-cold Tris-MgCl_2_ buffer supplemented with 1% PVP-10, 10 mM EDTA and 15 mM mercaptoethanol. The lysate was filtered through a 70 µM mesh filter and stained with 50 mg/mL propidium iodide and 50 mg/mL RNase solution [22,23]. Samples were analyzed using a BD FACSCanto II flow cytometer (Becton Dickinson and Company, Franklin Lakes, NJ, USA). The following voltages were used: 308 for FSC, 291 for SSC and 247 for PI. Debris background factor was set to 75,000. Data were processed by the FCSExpress7 software (DeNovo Software, Pasadena, CA, USA).

### 4.3. Genetic Analysis

DNA was extracted from fresh needles, homogenized in a Fastprep^®^-24 instrument (MP Biomedicals, Irvine, CA, USA), using the CTAB method [24].

ISSR analysis was performed using the primers (GTG)_5_, (CA)_6_GT and (AGC)_6_G according to the protocol described by Sboeva et al. [16,17] and visualized in 1.7% agarose gel. SSR analysis was done with primers Lar_eSSR11, Lar_eSSR54, Lar_eSSR69, Lar_eSSR78F, Lar_eSSR96, Lar_eSSR111, Lar_eSSR115, Lar_eSSR115, Lar_eSSR228, bcLK056, bcLK224, bcLK232, bcLK260 and bcLK235 (Table 2), recommended by Dong et al. and Kulakov et al. and visualized in 10% polyacrylamide gel [10,11].

Amplification and sequencing of the internal transcribed spacer (ITS) was performed according to Araki et al. using primers 5′-TGCGGTAGGATCATTGATAGCA-3′ and 5′-AGCCCAAACCTATCCATCCGA-3′ [9]. Amplification and sequencing of the trnT-trnF region and total trnK intron was done as described by Wei et al. and Bashalkhanov et al. with primers trnTF (5′-CATTACAAATGCGATGCTCT-3′), trnLR (5′-TCTACCGATTTCGCCATATC-3′), trnLF (5′-CGAAATCGGTAGACGCTACG-3′), trnFR (5′-TTTGAACTGGTGACACGAG-3′), trnKF (5′-AACCCGGAACTAGTCGGATG-3′) and trnRF (5′-GGTTGCGAGCTCAATGGTAGAGT-3′) [8,21]. Amplification of atpF-atpH, psbK-psbI, rbcL and rpoC1 markers was carried out according to Matveeva et al. [25]. Primers for amplification of *atpF-atpH* marker were 5′-ACTCGCACACACTCCCTTTCC-3′ and 5′-GCTTTTATGGAAGCTTTAACAAT-3′; for psbK-psbI marker—5′-TTAGCCTTTGTTTGGCAAG-3′ and 5′-AGAGTTTGAGAGTAAGCAT-3′; for rbcL marker—5′-GTAAAATCAAGTCCACCRCG-3′ and 5′-ATGTCACCACAAACAGAGACTAAAGC-3′ and for rpoC1 marker—5′-CCATAAGCATATCTTGAGTTGG-3′ and 5′-GGCAAAGAGGGAAGATTTCG-3′. 

*Picea abies* genes *mTERF* (MA_39589g0010) and *GIGANTEA* (MA_19575g0010) [12] were extracted from the Gymno PLAZA database. The search for homologous genes was carried out in whole-genome shotgun contigs of *L. sibirica* via NCBI BLAST tool. Alignment was performed in SnapGene software. Primers for the amplification of target genes were selected via the NCBI Primer designing tool. Primers 5′-TTGTTTTCAGAGGACCCAGC-3′ and 5′-ACCCATAGAGAATGATGGACCC-3′ were used for amplification and sequencing of the *mTERF* gene. Primers 5′-TCCCGCATGGCTGTTATCTA-3′ and 5′-CTTCTGCAACACGAGGGGTA-3′ were used for partial amplification and sequencing of the *GIGANTEA* gene. 

All amplifications were performed in the MiniAmp Plus Thermal Cycler (Thermo Fisher Scientific, USA), i.e., to initial denaturation at 95 °C for 5 min; followed by 35 cycles of 40 s denaturation at 94 °C, 40 s annealing at 56 °C, and 40 s elongation at 72 °C; with final elongation at 72 °C for 2 min. Products were purified with ExoSAP-IT PCR Product Cleanup Reagent (Thermo Fisher Scientific, Waltham, WA, USA), prepared using BigDye Terminator v3.1 Cycle Sequencing Kit and subjected to Sanger sequencing in Applied Biosystems 3500 genetic analyzer (Thermo Fisher Scientific, Waltham, WA, USA).

## Figures and Tables

**Table 1:** Analysis of banding pattern generated by the ISSR primer (GTG)_5_. Bands that can be used to identify the larches of Kuzhanovo are in bold.

Band Size, bp	Control Plants		Larches of Kuzhanovo			Distanced Larches		
1000	1	0	1	1	1	0	1	0
900	1	1	1	1	1	1	1	1
800	0	0	**1**	**1**	**1**	1	0	0
760	0	0	**1**	**1**	**1**	0	0	0
600	1	1	1	1	1	0	1	1
550	0	0	0	0	1	0	0	1
510	0	0	0	1	1	1	1	1
450	0	0	**1**	**1**	**1**	0	0	0
400	0	0	0	0	1	0	1	1

**Table 2:** List of SSR primers.

Primer Name	Forward Primer	Reverse Primer
Lar_eSSR11	5′-AATCCAAATTTCTGGACCCC-3′	5′-CCTGCAAAAAGAGGATAGCG-3′
Lar_eSSR54	5′-GCGCGCTCTTCTTTTCTCT-3′	5′-CGCCGTCGACTGTATAACCT-3′
Lar_eSSR69	5′-CAGCTGTAATGAATTCCGCA-3′	5′-GAAATGATGCAGGCAGAGGT-3′
Lar_eSSR78F	5′-CAATCCGATAAAACGCCATC-3′	5′-CAGTAACACTCCCGCCTAGC-3′
Lar_eSSR96	5′-GCCTTCGCTGATCTGTTTTC-3′	5′-TGCTGGTCTCTGTTGTCGTC-3′
Lar_eSSR111	5′-GATATCAACTCCCTGCGGAA-3′	5′-AGCTGTGAGCGAGAGAGAGG-3′
Lar_eSSR115	5′-TTGTGATGCTTCTTTGACCG-3′	5′-TTGTGATGCTTCTTTGACCG-3′
Lar_eSSR228	5′-CTCTCGTCCATTAAGCTGCC-3′	5′-GAGGATTGTGCACACCTTGA-3′
bcLK056	5′-ATGGGCTAAGGTATGTTTTACG-3′	5′-TGCCAACATCTATACCAAGTCT-3′
bcLK224	5′-GAGAGGCCACTACTATTATTAC-3′	5′-ATGCGTTCCTTCATTCCTCT-3′
bcLK232	5′-TGTTGCTGGGTTGTTGTTAGA-3′	5′-GGGTAATAGTTCCAGTCTTTG-3′
bcLK260	5′-CTCCATAAGGGGCATCACAT-3′	5′-TGGGCTCAAGTTTGGACATTA-3′
bcLK235	5′-TTCACTTGTGATCCTAGAGTTAGA-3′	5′-AACCCCTAACCATATAATATCCA-3′

## Data Availability

The sequences of the *atpF–atpH* intergenic spacer and *rpoC1* gene were submitted to the NCBI database (OP207953 and OP341614).
